# The Antioxidant Cyclic 3-Hydroxymelatonin Promotes the Growth and Flowering of *Arabidopsis thaliana*

**DOI:** 10.3390/antiox11061157

**Published:** 2022-06-13

**Authors:** Hyoung Yool Lee, Kyoungwhan Back

**Affiliations:** Department of Biotechnology, College of Agriculture and Life Sciences, Chonnam National University, Gwangju 61186, Korea; xanthine657@jnu.ac.kr

**Keywords:** antioxidant, cyclic 3-hydroxymelatonin, *Escherichia coli* expression, *FLOWERING LOCUS T*, gibberellin, melatonin, pathogen resistance, salt tolerance

## Abstract

In plants, melatonin is metabolized into several compounds, including the potent antioxidant cyclic 3-hydroxymelatonin (3-OHM). Melatonin 3-hydroxylase (M3H), a member of the 2-oxo-glutarate-dependent enzyme family, is responsible for 3-OHM biosynthesis. Although rice *M3H* has been cloned, its roles are unclear, and no homologs in other plant species have been characterized. Here, we cloned and characterized *Arabidopsis thaliana M3H* (*AtM3H*). The purified recombinant AtM3H exhibited *K*_m_ and *V*_max_ values of 100 μM and 20.7 nmol/min/mg protein, respectively. M3H was localized to the cytoplasm, and its expression peaked at night. Based on a 1,1-diphenyl-2-picrylhydrazyl (DPPH) assay, 3-OHM exhibited 15-fold higher antioxidant activity than melatonin. An Arabidopsis *M3H* knockout mutant (*m3h*) produced less 3-OHM than the wildtype (WT), thus reducing antioxidant activity and biomass and delaying flowering. These defects were caused by reduced expression of *FLOWERING LOCUS T* (*FT*) and gibberellin-related genes, which are responsible for flowering and growth. Exogenous 3-OHM, but not exogenous melatonin, induced *FT* expression. The peak of *M3H* expression at night matched the *FT* expression pattern. The WT and *m3h* exhibited similar responses to salt stress and pathogens. Collectively, our findings indicate that 3-OHM promotes growth and flowering in *Arabidopsis*.

## 1. Introduction

Plants produce many bioactive molecules pivotal for their growth, development, and adaptation to biotic and abiotic stresses [1]. Melatonin has multiple physiological activities at low concentrations, for which its interaction with the melatonin receptor is a prerequisite [2,3,4]. Melatonin-mediated pathogen resistance, chloroplast protein quality control, and endoplasmic reticulum stress tolerance are mediated by a mitogen-activated protein kinase signaling pathway in *Arabidopsis* [5,6,7,8]. Exogenous melatonin enhanced plant tolerance to biotic stresses such as a bacterium [9], fungus [10,11], and virus [12] and to abiotic stresses such as cold [13,14], drought [15,16], light [6], salt [17], and heavy metals [18,19], among others [20,21,22].

Melatonin is ubiquitous in plants with chloroplasts, likely because the ability to synthesize melatonin may derive from cyanobacteria, the precursors of chloroplasts [23,24]. Melatonin levels in healthy plant tissues range from picograms to a few nanograms per gram of fresh weight (FW) [25] but are increased several hundred-fold by stress [26]. The low melatonin level in plants may be attributed to the absence of the first two genes in melatonin biosynthesis—tryptophan decarboxylase (TDC) and tryptamine 5-hydroxylase (T5H), especially in *Arabidopsis* [25]. Other factors include the low activity of the last two enzymes in melatonin biosynthesis—serotonin *N*-acetyltransferase (SNAT) and *N*-acetylserotonin *O*-methyltransferase (ASMT)—and rapid melatonin metabolism [25].

In plants, melatonin 2-hydroxylase (M2H) converts melatonin into 2-hydroxymelatonin (2-OHM). *M2H*, a member of the 2-oxoglutarate-dependent dioxygenase (2-ODD) gene family, was cloned in rice [27]. In plants, exogenous 2-OHM conferred tolerance to combined cold and drought stress [28], as well as cadmium [29] stress. The second gene in plant melatonin metabolism is melatonin 3-hydroxylase (M3H), which converts melatonin into cyclic 3-hydroxymelatonin (3-OHM) [30]. M3H is a member of the 2-ODD family and shares only a 2-ODD domain with M2H. 3-OHM production peaks at night, and the overproduction induced by *M3H* overexpression increases the secondary tiller number in rice, indicating that 3-OHM functions at the reproduction stage of rice growth [31].

Homologs in other plant species of the above two melatonin metabolism-related genes have not been cloned or characterized. In this study, we cloned *Arabidopsis M3H* and characterized its activity. *M3H* knockout *Arabidopsis* (*m3h*) showed reduced growth and antioxidant activity in conjunction with delayed flowering, suggesting that 3-OHM promotes plant growth and reproduction.

## 2. Materials and Methods

### 2.1. Escherichia coli Expression and Purification of Recombinant Arabidopsis thaliana Melatonin 3-Hydroxylase (M3H)

A full-length *Arabidopsis thaliana M3H* (*AtM3H*) cDNA (AT1g17020) was kindly provided by the RIKEN BioResource Center (Ibaraki, Mito City, Japan) [32]. *AtM3H* was amplified by PCR using a specific primer set. The forward and reverse primers were 5′-AAA AAG CAG GCT CCA TGG AAG CAA AAG GGG-3′ and 5′-AGA AAG CTG GGT TTA GAT TCT CAA AGC ATC-3′, respectively. The first PCR product was diluted with water by 10-fold, and 1 μL aliquot was further amplified using a primer set harboring the *attB* recombination sequences (forward primer, 5′-GGG GAC AAG TTT GTA CAA AAA AGC AGG CT-3′; reverse primer, 5′-GGG GAC CAC TTT GTA CAA GAA AGC TGG GT-3′). The resulting AtM3H PCR products were gel-purified and cloned into the pDONR221 Gateway^®^ vector (Invitrogen, Carlsbad, CA, USA) using the BP (between the *attB* and the *attP* sites) recombination reaction. The pDONR221:AtM3H plasmid was then recombined with the pET300 Gateway destination plasmid through LR (between the *attL* and the sites) recombination to generate pET300-AtM3H, followed by transformation into *E. coli* BL21 (DE3) (Invitrogen). Bacterial culture and further affinity purification steps using a Ni-NTA column were performed according to the manufacturer’s instructions (Qiagen, Tokyo, Japan). Purified recombinant AtM3H protein was concentrated using an Ultrafree-4 centrifugal filter (Biomax-10, Millipore, Bedford, MA, USA), dissolved in 10 mM Tris-HCl (pH 8.0) containing 50% glycerol, and stored at −20 °C until further analysis.

### 2.2. Measurement of In Vitro M3H Activity and Enzyme Kinetics

Enzyme reaction medium contains 10 mM Tris/HCl (pH 8.0), 1 mM ascorbate, 100 µM FeSO_4_, 160 µM α-ketoglutarate, 100 µM melatonin, and 400-µg catalase. The reaction (100-µL) was incubated in the presence of the purified recombinant AtM3H protein at 30 °C for 1 h and stopped by adding 50-µL methanol. The 10 µL reaction products were analyzed using an HPLC system equipped with a fluorescence detector (Waters, Milford, MA). We employed an Atlantis C18 column (Waters; 3.9 × 150 mm) with a methanol gradient from 30 to 45% within 12 min and isocratic elution of 45% for 30 min at a flow rate of 0.25 mL/min. 3-OHM was detected with excitation at 280 nm and emission at 348 nm. Under these conditions, 3-OHM was eluted at 19 min. For detection of 2-OHM, aliquots were subjected to HPLC with a UV detector system (Waters) with a Sunfire C18 column (4.6 × 150 mm; Waters) by isocratic elution with 35% methanol in 0.3% trifluoroacetic acid for 25 min at a flow rate of 0.25 mL/min. All measurements were conducted in triplicate. The substrate affinity (*K*_m_) and maximum reaction rate (*V*_max_) values for AtM3H were measured using Lineweaver-Burk plots. Cyclic 3-hydroxymelatonin (3-OHM) was a kind gift from Dr. Czarnocki (Warsaw University, Warsaw, Poland). 2-Hydroxymelatonin (2-OHM) and melatonin were obtained from Toronto Research Chemicals (North York, ON, Canada) and Sigma Aldrich (St. Louis, MO, USA), respectively. Prohexadione-Ca, an inhibitor of 2-ODD enzymes, was obtained from Fluka (Buchs, Switzerland). Melatonin, 3-OHM, and 2-OHM were initially dissolved in 1% methanol.

### 2.3. Quantification of Cyclic 3-Hydroxymelatonin (3-OHM) from Arabidopsis

To quantify the 3-OHM from *Arabidopsis* tissues, frozen *Arabidopsis* samples (100 mg) were ground to a powder in liquid nitrogen using a Tissuelyser II (Qiagen, Tokyo, Japan) and extracted with 1 mL of chloroform. The chloroform extracts were evaporated until dry and dissolved in 100 µL of 40% MeOH. These samples were subjected to HPLC as described above. Quantification of 3-OHM was performed as described previously [30].

### 2.4. Measurement of Radical Scavenging Activity

The radical scavenging activity of 3-OHM was measured using the 1,1-diphenyl-2-picrylhydrazyl (DPPH) method as described previously [33]. In brief, *Arabidopsis* leaves (0.1 g) were extracted with 1 mL methanol and spun down for 5 min at 12,000× *g*. The resulting supernatants of methanolic extracts (0.1 mL) or standard compounds of 3-OHM, 2-OHM, and melatonin dissolved in methanol were mixed with 0.9 mL of 0.15 mM DPPH solution at 27 °C for 20 min. The control was prepared as above without any extract. Radical scavenging activity was calculated using the following numerical formula: % radical scavenging activity = (control optical density [OD] − sample OD)/control OD) × 100.

### 2.5. Arabidopsis Plants and Growth Conditions

*A. thaliana* (L.) Heynh. Columbia-0 wild type (WT) and an *M3H* knockout mutant (*m3h*) thereof were grown in plastic pots containing a commercial horticulture substrate (coco peat, 47%; peat moss, 35%; vermiculite, 10%; zeolite, 7% (FarmHannong, Seoul, Korea): perlite (SJ Company, Ulsan, Korea); 3:1 ratio). Plants were incubated in a growth room under a 14 h light and 10 h darkness photoperiod at 23 °C with 60% humidity and a photon flux density of 50 μmol m^−2^ s^−1^. Each pot was covered with transparent film for 4 days to prevent water loss. Plants were illuminated with fluorescent light from OSRAM (Seoul, Korea) at 6500 K (865 FPL36EX-D) and 4000 K (840 FPL 36EX-W) at a 50:50 ratio. The *m3h* mutant (SALK-144842) was obtained from the Arabidopsis Biological Resource Center (ABRC, Columbus, OH, USA) and had a T-DNA insertion in the second exon from the 5′-end of *M3H* (AT1G17020). To confirm the mutant line as homozygous, PCR screening was conducted using the genomic DNA of *m3h* and gene-specific primers (forward, 5′-ATG GAA GCA AAA GGG GCA GC-3′; reverse, 5′-GAT TCT CAA AGC ATC TAG AT-3′).

### 2.6. Determination of Flowering Time

Flowering times were defined based on the days to bolting and the total number of rosette leaves after the main stem had bolted by 1 cm. For each replicate, the flowering time and number of rosette leaves were recorded per genotype. Leaf area was measured using Fuji ImageJ software [34]. Rosette leaves were photographed adjacent to a ruler; pixels were converted into metric units. Data were processed using Microsoft Excel 2010 (Microsoft Corp., Redmond, WA, USA) and are the means of three replicates from three independent plants.

### 2.7. Subcellular Localization Analysis of Arabidopsis thaliana Melatonin 3-Hydroxylase (AtM3H)

The pER8-mCherry for transgene localization analysis and pBIN61-GFP-HA (P35s:GFP-HA) plasmid for the cytoplasmic fluorescence marker were kindly provided by Dr. HG Kang (Texas state university, San Marcos, TX, USA). As for the subcellular localization study of *AtM3H*, a full-length *AtM3H* cDNA was amplified with PCR using a primer set containing an *AscI* site (forward, 5′-GGC GCG CCA TGG AAG CAA AAG GGG CAGC-3′, reverse, 5′-GGC GCG CCG GAT TCT CAA AGC ATC TAG-3′). The amplified PCR product was first introduced into the T&A vector (RBC Bioscience, New Taipei City, Taiwan), from which the *AscI* insert of *AtM3H* was prepared and ligated into the *AscI* site of the binary vector pER8-mCherry to generate pER8-AtM3H-mCherry. The resulting plasmids were transformed into the *Agrobacterium Tumefaciens* strain GV2260 using the freeze-thaw method. Leaves from four-week-old tobacco (*Nicotiana benthamiana*) plants, native Australian species, were infiltrated with *Agrobacterium* strain (about 0.2 OD), followed by infiltration with 10 μM β-estradiol, and then incubated for 24 h in a growth room. Images were obtained using a Leica TCS-SP5 confocal microscope (Leica, Wetzlar, Germany) using a ×63 oil-immersion objective. Images were processed using the Leica LAS-AF software version 1.8.2 (Leica, Wetzlar, Germany).

### 2.8. RNA Extraction and Gene Expression Analysis

For various chemical-induced gene expression analyses, leaves of five-week-old *Arabidopsis* plants were syringe infiltrated with 4 µM of various chemicals in buffer containing 2 mM MgCl_2_ in 5 mM MES of pH 5.7 under regular dark-light conditions, and leaf samples were collected 2 and 4 h after infiltration. A control solution was 0.001% methanol in 2 mM MgCl_2_ in 5 mM MES (pH 5.7). Total RNA was extracted using a Nucleospin RNA Plant Kit (Macherey-Nagel, Duren, Germany). The RNA (800 ng) was used to prepare cDNA using the RNA to cDNA EcoDry^TM^ premix system (TaKaRa Biotechniques, Shiga, Japan). The cDNA was diluted 2-fold, and 1-uL was used as a template in each reverse transcription-polymerase chain reaction (RT-PCR) analysis. The PCR reaction was conducted under the following conditions, with initial denaturation at 95 °C (3 min), denaturation at 95 °C (30 s), annealing at 56 °C (30 s), and extension at 72 °C (1 min) in a total of 30-uL of the reaction mix. The primer sequences for RNA analysis were as follows: *FT* (forward, 5′-GGT GGA GAA GAC CTC AGG AA-3′, reverse, 5′-CTC ATT TTC CTC CCC CTC TC-3′); *KS* (forward, 5′-CCA AGT TGA TCT GGC AGG TA-3′, reverse, 5′-TTG TCT CCT AAA ATC AAT TTT CCT C-3′); *GA3ox1* (forward, 5′-AGA GTG TGG GAG GTG AAT GG-3′, reverse, 5′-TTT TGC CTC CTC TTG GTC TC-3′); *GA3ox2* (forward, 5′-CCC ATC TCT CAC TTG GAA ACA-3′, reverse, 5′-TTG TGA ATT TGA AGA ACC ACT CA-3′); *MYB33* (forward, 5′-TTG TTC TTG GAG CAA CAT GC -3′, reverse, 5′-TGC ATT GGC AGT TGC TAG TC-3′); *M3H* (forward, 5′-GAA GCA AAA GGG GCA GC-3′, reverse, 5′-TCT CAA AGC ATC TAG AT-3′). *EF-1a* was used for signal normalization (forward, 5′-TGG TGA CGC TGG TAT GGT TA-3′, reverse 5′-CAT CAT TTG GCA CCC TTC TT-3′).

### 2.9. Bacterial Growth Assay

The bacterial strain used in this study was virulent *Pseudomonas syringae* pv. Tomato DC3000 (*Pst* DC3000) strain and the avirulent avrRpm1-containing *Pst* DC3000 strain (*Pst*-avrRpm1) were obtained from Dr. CS Oh (Kyunghee University, Yongin, Korea). Bacterial culture was grown in King’s medium B in the presence of 50 µg rifampicin and 50 µg kanamycin per milliliter. Five- to six-week-old mature plants were spray inoculated with bacterial solutions containing 5 × 10^6^ CFU/mL or 1 × 10^8^ CFU/mL (in 0.01% [*v*/*v*] Silwet L-77) for *Pst* DC3000 or *Pst-*avrRpm1, respectively. Bacterial titers were assayed at 5 d after spray inoculation. For each measurement, extracts from 7 leaf discs (from three plants) were serially diluted 10 times, then 10 µL of each dilution was plated on King’s medium B agar plates containing both 50 µg rifampicin and 50 µg kanamycin per milliliter. Each data point represents three replicates.

### 2.10. Salt Tolerance Assay

To determine tolerance to salt stress, at least 30 seeds of the WT and *m3h* per experiment were sown on MS, without or with 100 mM NaCl. To evaluate primary root growth under salt stress, seeds on MS medium were incubated vertically in a growth room for 3 weeks. Primary roots were photographed, and their lengths were measured using Fuji ImageJ software [34].

### 2.11. Statistical Analysis

The data were analyzed by analysis of variance using IBM SPSS Statistics 25 software (IBM Corp. Armonk, NY, USA). Means with different letters or asterisks indicate significantly different values at *p* < 0.05 according to a post-hoc Tukey’s honest significant difference (HSD) test. All data are presented as mean ± standard deviation.

## 3. Results

### 3.1. Sequence Analysis of Arabidopsis and Rice M3H

**Figure 1:**
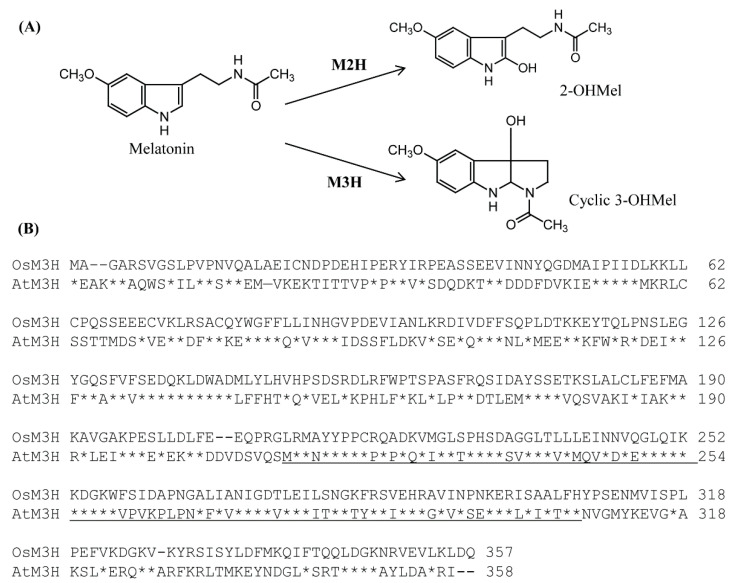
Reaction catalyzed by M3H and amino acid sequences of rice OsM3H and *Arabidopsis* AtM3H. (**A**) Enzymatic conversion of M2H and M3H. (**B**) Amino acid sequences of OsM3H and AtM3H. Asterisks, identical amino acids; dashes, gaps; underlining, conserved 2-ODD domain. GenBank accession numbers of *OsM3H* and *AtM3H*, AK067086 and AT1g17020, respectively.

A BLAST search [35] using rice (*Oryza sativa*) *M3H* (AK067086) [30] as a query led to the identification of one *M3H-*like gene in *Arabidopsis thaliana* (*AtM3H;* AT1g17020). *AtM3H* encoded a protein of 358 amino acid residues, and the AtM3H polypeptide shared a 40% amino acid identity with rice M3H (Figure 1). Similar to rice M3H, neither chloroplast nor mitochondria transit peptide was present in AtM3H according to TargetP analysis [36], suggesting a cytoplasmic protein. Notably, the 2-ODD conserved domains of the OsM3H and AtM3H polypeptides showed 60% homology. *Arabidopsis AtM3H* is the sole homolog of rice *M3H*.

### 3.2. M3H Activity of Recombinant AtM3H

**Figure 2:**
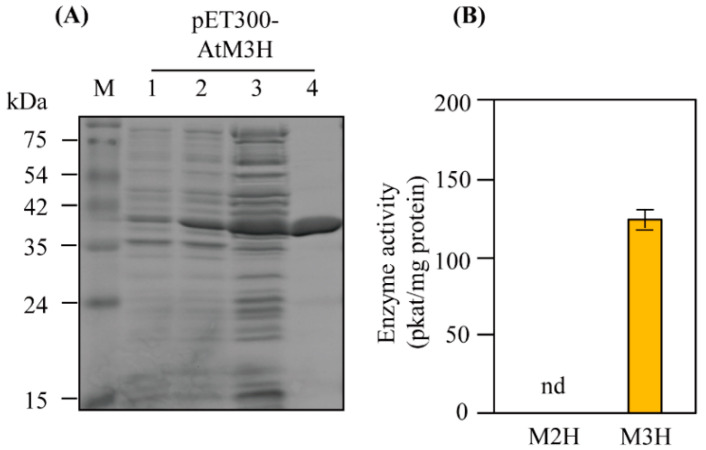
Affinity purification of His-tagged *Arabidopsis* AtM3H and M3H and M2H activities. (**A**) Expression and affinity purification of His-tagged AtM3H from *E. coli*. (**B**) Specific activities of M3H and M2H. Proteins were resolved by SDS-PAGE followed by Coomassie blue staining. M, molecular standard; lane 1, total protein in 10 µL aliquots of bacterial suspension without IPTG; lane 2, total protein after IPTG induction; lane 3, 10 µg of protein in the supernatant after centrifugation at 10,000× *g*; lane 4, AtM3H protein (10 µg) purified by affinity (Ni-NTA) chromatography.

**Figure 3:**
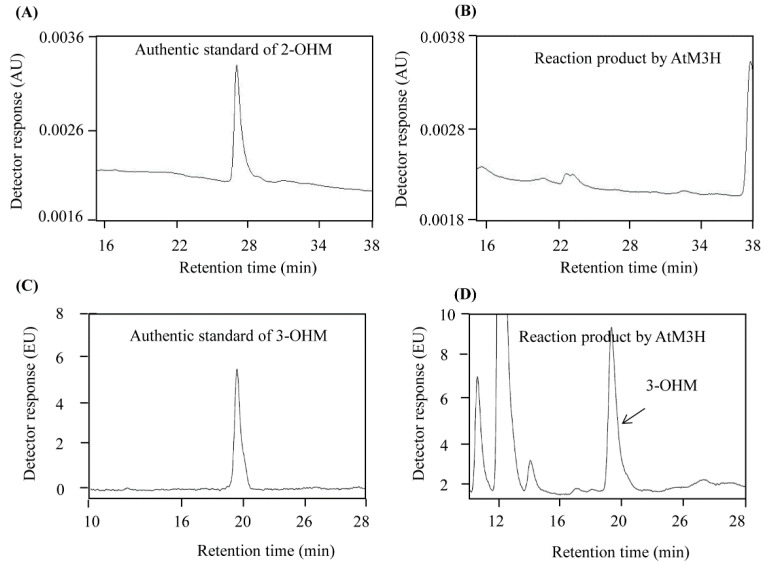
HPLC of in vitro products of purified recombinant AtM3H. (**A**) 2-OHM authentic compound. (**B**) In vitro product of AtM3H. (**C**) 3-OHM authentic compound. (**D**) In vitro product of AtM3H. EU, emission units; AU, absorption units.

Due to the low homology (40%) between OsM3H and AtM3H, we examined whether AtM3H has M3H activity. Full-length *AtM3H* was expressed in-frame with the coding sequence of the amino-terminal hexahistidine in *Escherichia coli* and induced by IPTG (Figure 2A). Soluble recombinant AtM3H was purified using a nickel affinity column. The recombinant proteins had 98% homogeneity, as determined by sodium dodecyl sulfate-polyacrylamide gel electrophoresis (SDS-PAGE) and Coomassie staining. AtM3H exhibited M3H activity of 125 pkat/mg protein but no M2H activity (Figure 2B). By contrast, rice OsM3H showed M3H and M2H activities, although its M2H activity was low [30]. Chromatograms of 2-OHM and 3-OHM authentic compounds, and AtM3H reaction products, are shown in Figure 3.

### 3.3. Characteristics of Recombinant AtM3H

**Figure 4:**
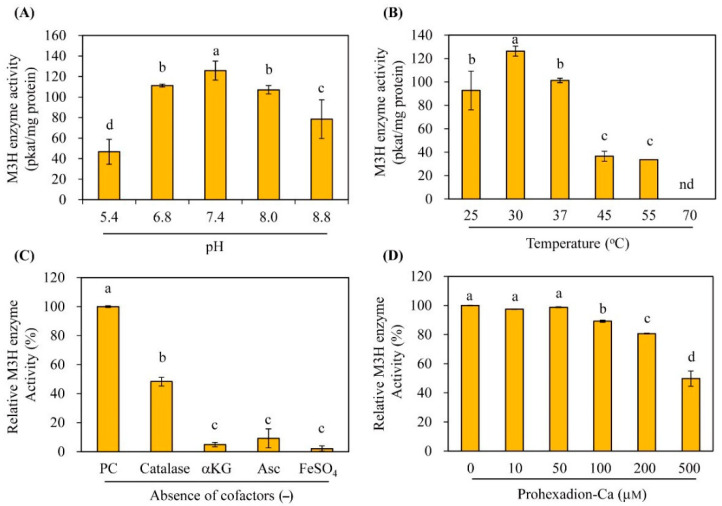
Kinetics of purified AtM3H. M3H activity as a function of (**A**) pH and (**B**) temperature. (**C**) Effects of cofactors on M3H activity. (**D**) Effect of prohexadione-Ca on M3H activity. Means ± SD of three replicates. Different letters (a–d) indicate significant differences at *p* < 0.05. PC, M3H activity with all cofactors; α-KG, α-ketoglutarate; Asc, ascorbic acid; nd, not detected.

**Figure 5:**
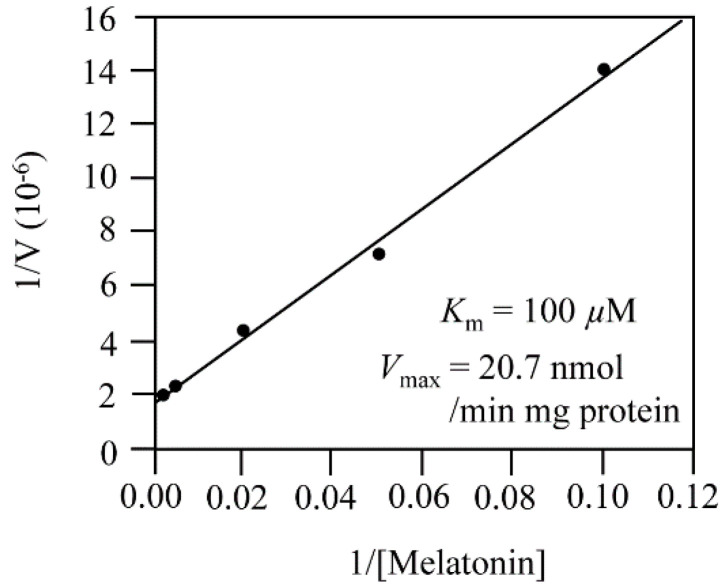
Enzyme kinetic analysis. *V*_max_ and *K*_m_ based on Lineweaver–Burk plots.

The optimum pH of M3H was pH 7.3, but M3H activity was high at pH 6.8–8.8 (Figure 4). The optimum temperature was 30 °C, followed by 37 °C and 25 °C; activity was absent at 70 °C. The optimum pH and temperature of AtM3H are similar to those of rice OsM3H, which also exhibited the optimum pH (7.4–8.0) and temperature (30 °C) [27,30]. AtM3H required α-ketoglutarate as a cosubstrate and several cofactors (ascorbate, Fe^2+^, and catalase); the absence of these factors markedly reduced or abolished AtM3H activity (Figure 4). At 100 and 500 μM, the 2-ODD inhibitor prohexadione-Ca decreased AtM3H activity by 10% and 50%, respectively. By contrast, 10 μM prohexadione-Ca reduced OsM2H activity by 90% [27]. Therefore, AtM3H was more tolerant of prohexadione-Ca than M2H (Figure 4D). Using the above optimum conditions, the *K*_m_ and *V*_max_ values of AtM3H were 100 μM and 20.7 nmol/min mg protein, respectively (Figure 5). The *K*_m_ of AtM3H was sevenfold lower than that of OsM3H, whereas the *V*_max_ was 10-fold lower. The catalytic efficiency (*V*_max_/*K*_m_) of AtM3H was 1.3-fold lower than that of OsM3H. Therefore, AtM3H is a member of the 2-ODD superfamily and has a catalytic efficiency similar to rice OsM3H.

### 3.4. Subcellular Localization of AtM3H

**Figure 6:**
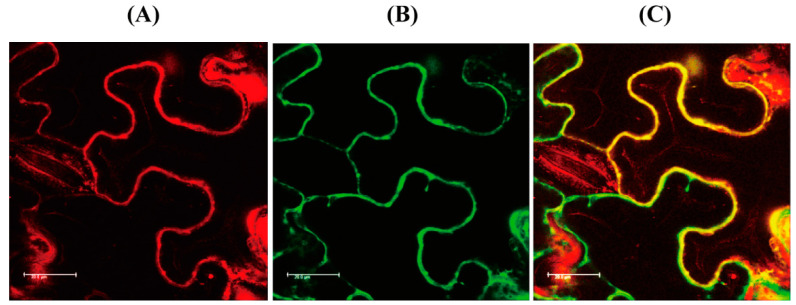
Subcellular localization. (**A**) Red fluorescence of AtM3H-mCherry. (**B**) Green fluorescence of cytoplasmic GFP-HA. (**C**) Merged fluorescence image of A and B. Tobacco leaves were infiltrated with *Agrobacterium tumefaciens* (GV2260) containing XVE-inducible AtM3H-mCherry or constitutive 35S promoter-driven GFP-HA (cytoplasm marker). Bars, 20 μm.

TargetP analysis predicted that AtM3H is localized to the cytoplasm due to the absence of transit sequences. AtM3H-mCherry fusion protein was transiently induced in tobacco leaves and observed by confocal microscopy. The red fluorescence of AtM3H-mCherry colocalized with the green fluorescence of green fluorescent protein (GFP) in the cytoplasm, suggesting that AtM3H is localized to the cytoplasm as is rice M3H (Figure 6).

### 3.5. The Knockout Mutant of M3H (m3h) had Low Antioxidant Activity

**Figure 7:**
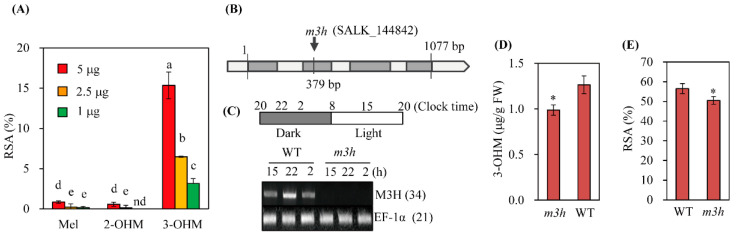
Schematic diagram of the genome structure and antioxidant activity of *m3h*. (**A**) Antioxidant activities of melatonin, 2-OHM, and 3-OHM revealed by DPPH assay. (**B**) Schematic of the T-DNA insertion site (downward arrow) in *M3H*. Gray and white boxes indicate exons and introns, respectively (not to scale). (**C**) Expression of *M3H* in the WT and *m3h* revealed by RT-PCR over 24 h. Numbers in parentheses are numbers of PCR cycles. *Elongation factor-1 alpha* (*EF-1α*) (AT5g60390) served as the loading control. (**D**) 3-OHM levels in leaves of the WT and *m3h* infiltrated with melatonin (100 µM). (**E**) Antioxidant activities of lower leaves of 30-day-old WT and *m3h*, as revealed by DPPH assay. Error bars are standard deviations of three biological replicates. Different letters and asterisks (*) indicate significant differences (Tukey’s post hoc HSD test; *p* < 0.05). RSA, radical scavenging activity.

3-OHM exerts an antioxidant effect by scavenging hydroxyl radical (·OH) and peroxyl radical (·OOH) [37,38]. A 1,1-diphenyl-2-picrylhydrazyl (DPPH) assay showed (Figure 7A) that 3-OHM exhibited 15-fold higher activity than melatonin; 2-OHM showed the lowest antioxidant activity, as reported previously [39]. *m3h* did not express *M3H* over 24 h, whereas the WT exhibited peak *M3H* expression at 10 pm (4 h after the light-to-dark transition) and declined thereafter to the level observed under light conditions (Figure 7B,C). Therefore, the *Arabidopsis M3H* transcript level shows a diurnal rhythm that peaks at night, consistent with rice *M3H* [31]. Due to the lack of *M3H* expression, *m3h* produced less 3-OHM than the WT (Figure 7D), resulting in lower leaf antioxidant activity (Figure 7E). The moderate 3-OHM level in *m3h* suggests the presence of several *M3H*-like genes in *Arabidopsis*, as in rice [30].

### 3.6. The m3h had Delayed Flowering and Retarded Growth

**Figure 8:**
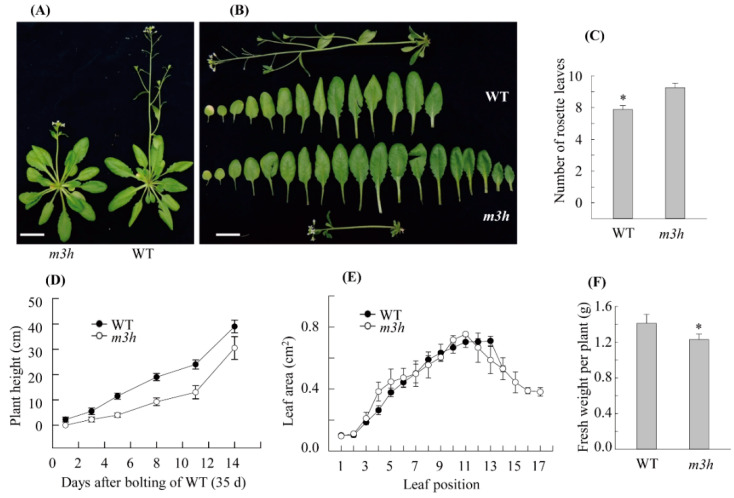
Phenotypic characterization of *m3h*. (**A**) Representative flowering phenotypes. (**B**) Rosette leaf phenotypes. (**C**) Flowering times of the WT and *m3h*, measured as the number of rosette leaves at bolting. (**D**) Time course of plant height after bolting of the WT and *m3h*. (**E**) Leaf area measurements by leaf position. (**F**) Fresh weight of the WT and *m3h* at bolting. *Arabidopsis* plants were grown under a 14 h light/10 h dark cycle and photographed 4–6 days after bolting (39 days old). Scale bars, 2 cm. Error bars are standard deviations of three biological replicates. Asterisks (*) indicate significant differences (Tukey’s post hoc HSD test; *p* < 0.05).

**Figure 9:**
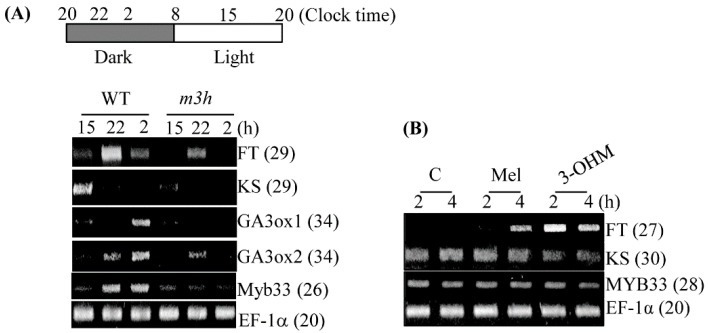
Expression profiles of flowering-related genes in the WT and *m3h*. (**A**) Daily expression of *FLOWERING LOCUS T* (*FT*) and gibberellin (GA)-related genes in the WT and *m3h*. (**B**) Expression levels of *FT*, *KS*, and *MYB33* in WT leaves in response to melatonin and 3-OHM (4 μM). Transcript levels were analyzed by RT-PCR using cDNA from leaves of 30-day-old mature plants before the bolting stage. Plants were sampled at the indicated times. *EF-1α* was used as the loading control. Numbers in parentheses are numbers of PCR cycles. Effector solutions were infiltrated into the abaxial leaf side at 8 pm and incubated for 2 and 4 h in darkness. Accession numbers: *FT*, AT1G65480; *KS*, AT1g15550; *GA3ox1*, AT1g80340; *GA3ox2*, AT5g06100; *MYB33*, AT5g60390; and *EF-1α*, AT5g60390.

We next investigated the growth and development of WT and *m3h* plants. *m3h* plants were smaller than the WT and possessed more rosette leaves, indicating delayed flowering (Figure 8). In addition, the total biomass per plant of *m3h* was lower than the WT. The stunted growth was caused by decreased expression of the gibberellin (GA) biosynthesis-related genes *ent-kaurene synthase* (*KS*), *GA3 oxidase1* (*GA3ox1*), and *GA3 oxidase2* (*GA3ox2*) (Figure 9). The expression of the GA-responsive positive transcription factor *Myb33* was also decreased. Regarding delayed flowering, the expression of the key flowering inducer, *FLOWERING LOCUS T* (*FT*), was decreased in *m3h* compared to the WT. Unlike *KS*, *FT* expression was rapidly and significantly induced by exogenous 3-OHM, and to a lesser degree by melatonin. Therefore, 3-OHM–mediated suppression of *FT* expression contributes to the delayed flowering in *m3h*, as does decreased GA synthesis.

### 3.7. Stress Tolerance of m3h

**Figure 10:**
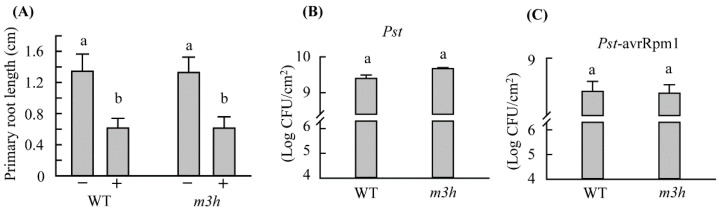
Stress responses of the WT and *m3h*. (**A**) Root length in response to salt stress. Seeds were sown on MS medium in the absence (−) and presence (+) of 100 mM NaCl and vertically positioned for 3 weeks. (**B**) Bacterial growth in response to virulent *P. syringae* pv. tomato DC3000 (*Pst*). (**C**) Bacterial growth in response to avirulent *P. syringae* pv. tomato DC3000 containing avrRpm1 (*Pst*-Rpm1). Bacteria were spray-inoculated at 5 × 10^6^ (*Pst*) or 1 × 10^8^ CFU/mL (*Pst*-Rpm1) (in 0.01% [*v*/*v*] Silwet L-77), and their growth was determined at 5 days post-infection. Experiments were conducted in triplicate. Different letters indicate significant differences (Tukey’s post hoc HSD test; *p* < 0.05). CFU, colony-forming units.

The *m3h* and WT *Arabidopsis* seeds were sown on an MS medium containing 100 mM NaCl for 3 weeks. There was no difference in root length between the WT and *m3h*, indicating that a decreased 3-OHM level is not significantly associated with the response to salt stress (Figure 10A). A virulent pathogen (*Pseudomonas syringae* pv. tomato DC3000) and its avirulent variant containing avrRpm1 (Pst-avrRpm1) were used to evaluate pathogen resistance. Leaves of *m3h* and the WT exhibited similar responses to the two pathogens (Figure 10B,C), indicating that 3-OHM is not implicated in the plant response to pathogen infection. These data were in contrast with those of the *snat1* knockout mutant, which showed the increased susceptibility to the avirulent pathogen of Pst-avrRpm1 [40], indicative of the different role of 3-OHM compared to melatonin concerning pathogen response.

## 4. Discussion

In animals, melatonin is rapidly metabolized into various metabolites, including 6-hydroxymelatonin, by cytochrome P450 enzymes, followed by 6-sulfatoxymelatonin for urinary excretion [41]. Other animal melatonin metabolites include 3-OHM, 2-OHM, 4-hydroxymelatonin, *N*^1^-acetyl-*N*^2^-formyl-5-methoxykynuramine (AFMK), and *N*^1^-acetyl-5-methoxykynuramine (AMK). The first reported melatonin metabolite in plants was AFMK, the level of which in water hyacinth peaked during the late light phase; AFMK may be useful for phytoremediation of toxic chemicals or pollutants [42]. The first *M2H* gene in plant melatonin metabolism from the 2-ODD family was cloned from the rice genome [27]. Compared to SNAT, M2H has a lower *K*_m_ and higher *V*_max_ (145 μM and 18.5 pkat/mg protein, respectively). Consequently, the 2-OHM levels are higher than melatonin in numerous plant species [43,44]. *M3H*, also a member of the 2-ODD family, was cloned from the rice genome [30]. The catalytic efficiency (*V*_max_/*K*_m_) of M3H was higher than that of M2H, and the 3-OHM level was fourfold higher than the 2-OHM level in rice leaves challenged with 1 mM melatonin for 24 h. These data suggest that, in plants, melatonin is rapidly metabolized to 3-OHM followed by 2-OHM. Also, melatonin triggered AFMK and AMK production in rice seedlings. Surprisingly, neither 6-OHM nor 4-OHM was detected in rice seedlings, suggesting the difference of melatonin catabolic pathway between plants and animals [30].

2-OHM conferred resistance to cold-plus-drought stress on rice, tobacco, tomato, and cucumber [28,45]. Also, 2-OHM mediates the response to cadmium of cucumber by increasing the levels of antioxidants such as glutathione, and antioxidant enzymes such as superoxide dismutase [29,46]. 2-OHM induced reactive oxygen species (ROS) production, thereby accelerating leaf senescence [47] and promoting seed germination [48]. Conversely, *M2H* suppression in rice by RNAi enhanced melatonin production and salt tolerance, indicating that melatonin catabolism regulates the endogenous melatonin level in rice [49]. By contrast, the functions of 3-OHM in plants are unclear, although it reportedly scavenges radicals [37] and peroxyl radicals [38].

We report for the first time that 3-OHM has different functions than melatonin and 2-OHM in *Arabidopsis*. The lower *K*_m_ value of AtM3H than rice M3H reflected the lower melatonin level in *Arabidopsis* than in rice. The catalytic efficiency (*V*_max_/*K*_m_) of AtM3H was comparable to that of rice M3H. Suppression of rice *M3H* expression did not significantly affect growth, but decreased biomass and grain yield, thus implicating 3-OHM in growth and reproduction in rice [31]. The *Arabidopsis m3h* knockout mutant showed similar responses to salt and pathogen stresses compared to the WT, but exhibited lower antioxidant activity, delayed flowering, and stunted growth. A vitamin C-deficient *Arabidopsis* mutant showed early flowering [50], and 3-OHM deficiency delayed flowering in *m3h*. Delayed flowering was caused by the suppression of *FT* expression because 3-OHM is positively associated with *FT* expression. Although ascorbic acid and 3-OHM are antioxidants, they have different biological functions in flowering. Further studies of melatonin and its metabolites (e.g., 2-OHM and 3-OHM) will shed light on their biological functions and interactions in plants [7,25,46,51,52,53,54].

## 5. Conclusions

3-OHM is a potent antioxidant in vitro, but its physiological function in plants was previously unclear. In this study, *Arabidopsis M3H* had M3H activity in vitro. Furthermore, an *Arabidopsis M3H* knockout mutant (*m3h*) exhibited lower 3-OHM production than the WT, reducing leaf antioxidant activity. The *m3h* mutant also showed delayed flowering and less biomass than the WT. We report for the first time that 3-OHM is not only a potent antioxidant but also a signaling molecule that induces *FT* expression in vitro and in vivo, regulating flowering in *Arabidopsis*.

## Data Availability

Data presented in this study are available within the article.
